# Cryogenic Mechanical Properties and Stability of Polymer Films for Liquid Oxygen Hoses

**DOI:** 10.3390/polym15163423

**Published:** 2023-08-16

**Authors:** Yunguang Cui, Jia Yan, Juanzi Li, Duo Chen, Zhenyu Wang, Wenxuan Yin, Zhanjun Wu

**Affiliations:** 1 State Key Laboratory of Structural Analysis for Industrial Equipment, School of Aeronautics and Astronautics, Faculty of Vehicle Engineering and Mechanics, Dalian University of Technology, Dalian 116024, China; 2 School of Materials Science and Engineering, Dalian University of Technology, Dalian 116024, China

**Keywords:** polymer thin films, cryogenic mechanical properties, liquid oxygen compatibility, liquid oxygen composite hose

## Abstract

To select the appropriate polymer thin films for liquid oxygen composite hoses, the liquid oxygen compatibility and the cryogenic mechanical properties of four fluoropolymer films (PCTFE, ETFE, FEP and PFA) and two non-fluoropolymer films (PET and PI) before and after immersion in liquid oxygen for an extended time were investigated. The results indicated that the four fluoropolymers were compatible with liquid oxygen before and after immersion for 60 days, and the two non-fluoropolymers were not compatible with liquid oxygen. In addition, the cryogenic mechanical properties of these polymer films underwent changes with the immersion time, and the changes in the non-fluoropolymer films were more pronounced. The cryogenic mechanical properties of the two non-fluoropolymer films were always superior to those of the four fluoropolymer films during the immersion. Further analysis indicated that the fundamental reason for these changes in the cryogenic mechanical properties was the variation in the crystalline phase structure caused by the ultra-low temperature, which was not related to the strong oxidizing properties of the liquid oxygen. Analytical results can provide useful guidance on how to select the appropriate material combination to obtain a reasonable liquid oxygen composite hose structure.

## 1. Introduction

**Figure 1:**
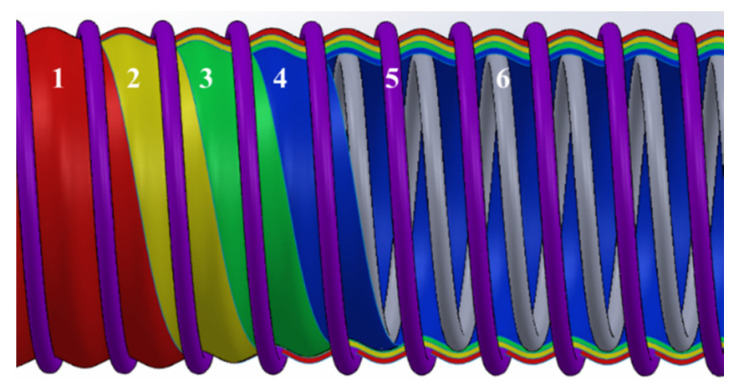
Structure of cryogenic composite hoses, including polymer film winding layers (1, 2, 3, 4) and inner and outer steel wires (5, 6).

With the continuous development of cryogenic technology, cryogenic composite hoses are increasingly being used to transport cryogenic media [1,2]. Cryogenic composite hoses are flexible pipes that are mainly wound by multi-layer polymer film materials [3], as shown in Figure 1. Cryogenic composite hoses, with their good bending performance, light weight and strong adaptability, are mostly used for transporting liquefied natural gas (LNG, −162 °C) [4], bringing significant value to the LNG industry [5,6]. The successful application of cryogenic composite hoses in the field of LNG transportation also provides a reference for the transportation of liquid oxygen (LOX, −183 °C). LOX has important application value in the fields of aerospace, medical treatments, submarines and others [7,8]. Especially in the field of aerospace, LOX is usually used as a propellant, together with other reducing agents [9]. However, the materials used in cryogenic composite hoses must be determined by the properties of the medium being transported; therefore, the selection of materials is the first critical step in the design and development of liquid oxygen hoses [10]. LOX is known as a strong oxidant with an ultra-low boiling point temperature (90 K) [11]. Therefore, it is necessary to investigate the performance and behavior of the polymer film materials used in liquid oxygen hoses, such as their compatibility with LOX, their cryogenic mechanical properties and their performance stability.

Liquid oxygen compatibility (LOC) is key to evaluate whether polymers and their composites can be used in a liquid oxygen environment. Currently, 98 J of mechanical shock in liquid oxygen ambient (ASTM G86-17) is commonly used to evaluate the liquid oxygen compatibility of materials [12,13,14]. There have been many reports about using this method to verify the liquid oxygen compatibility of polymer materials. DeLong et al. [15] showed that perfluoroalkoxy polymers (PFA) passed the 98 J LOC test, and they completed the manufacture and evaluation test for a fluoropolymer skin tank cylinder. Furthermore, some fluoropolymers, such as polytetrafluoroethylene (PTFE), polychlorotrifluoroethylene (PCTFE) and ethylene tetrafluoroethylene copolymer (ETFE), are commonly used as sealing materials for liquid oxygen pipe fittings [16,17,18], which means that these fluoropolymers passed the LOC test and are compatible with LOX. In addition to LOC, understanding the cryogenic mechanical behavior of a specialized set of polymer films is also important for their application under extremely low-temperature conditions [19].

Ross et al. [20] believed that lower-crystallinity samples of PCTFE showed a higher tensile modulus, yield stress, stress at failure and elongation to failure than higher-crystalline samples at 78 K and 4.2 K. Enzo [21] found that the cryogenic modulus and strength of fluoropolymers increased with decreasing temperature when the crystallinity was consistent. Other scholars have found that prolonged exposure to ultra-low temperatures has an impact on the cryogenic mechanical properties of polymers [22,23]. Indumathi et al. [24] found that after treating polyimide (PI), polyetherimide (PEI) and PTFE with liquid nitrogen (LN_2_, 77 K) for one day, the crystallinity of the two non-fluoropolymers increased, thereby improving their strength, but the crystallinity of PTFE did not significantly change. In addition, for semicrystalline polymers, the degree of crystallinity and grain size exert an effect on their cryogenic mechanical properties [25,26,27]. Rogere et al. [28] found that the tensile strength of polyethylene terephthalate (PET) films decreased with increasing crystallinity at liquid nitrogen and liquid hydrogen temperatures. Yano et al. [29] and Zhou et al. [30] also found that the tensile strength of PET and polyethylene-2,6-naphthalenedicarboxylate (PEN) films, whose chemical structures are similar to PET, decreased with increasing crystallinity at 77 K. Shao et al. [31] believed that after cryogenic treatment, the crystallinity and hardness of the polymer would be improved.

**Figure 2:**
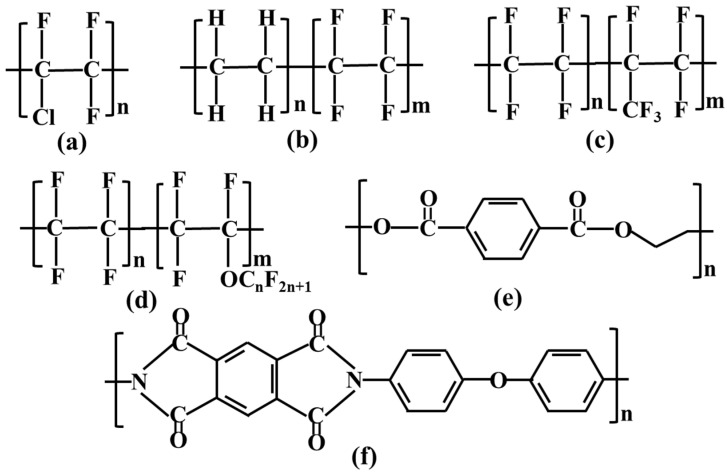
Molecular structure of PCTFE (**a**), ETFE (**b**), FEP (**c**), PFA (**d**), PET (**e**) and PI (**f**).

In recent years, most research work related to polymers for cryogenic engineering have focused on either mechanical properties or LOC, while the integrated consideration of their LOC, cryogenic mechanical properties and practical applications has rarely been explored. Therefore, in this paper, the cryogenic mechanical properties and LOC of four fluorinated polymers, PCTFE, PFA, perfluoroethylene propylene (FEP) and ETFE, as well as two non-fluoropolymers (PET and PI), were investigated. The PI films were sprayed with FEP powder. The emphasis was on the effects of the strong oxidation and ultra-low-temperature properties of LOX on the cryogenic mechanical properties of polymers. The structure of these polymer materials is shown in Figure 2. Through the research of this paper, the mechanical properties of several polymer materials at −196 °C were clarified, which accumulated valuable data for the application of polymer materials in ultra-low-temperature environments. In addition, the effect of the liquid oxygen environment on the properties of these polymer materials was further investigated, and the mechanism of the mechanical properties of polymer materials in an extended-time liquid oxygen environment was revealed, which provided basic data support for the research and development of liquid oxygen cryogenic composite hoses. Meanwhile, it was also of great significance for the research of other non-metallic liquid oxygen containers.

## 2. Experimental Section

### 2.1. Materials

All polymer film materials used in this study were commercial-grade. PCTFE was obtained from Honeywell Company (Morristown, NJ, USA), with a thickness of 0.05 mm; PFA and FEP were purchased from Chemours Company (Wilmington, DE, USA), with thicknesses of 0.05 mm and 0.035 mm, respectively; ETFE, PET and PI were provided by Guangdong Tyne Co., Ltd. (Heshan, Guangdong, China), with thicknesses of 0.15 mm, 0.025 mm and 0.05 mm, respectively.

### 2.2. Sample Preparation and Treatment

During the molding process of polymer films, two directions named the machine direction (MD) and the transverse direction (TD) are usually used to indicate the forming direction. In this study, in order to better compare the mechanical properties, we uniformly used MD as the tensile direction of samples. All specimens were cut according to the ASTM D638 Standard using a custom cutter with a sharp blade [32]. As mentioned in ASTM D638, the rectangular sample is easy to make and can effectively avoid the damage of sample edges. Therefore, the rectangular type was used as the shape for all specimens. The edges of these rectangular samples were checked, and the samples with damaged edges were discarded.

After all specimens were prepared, some were immersed in LOX for 7 days, 30 days and 60 days, respectively, and each group had at least five parallel specimens. In addition, every material left one group untreated (immersed for 0 days) as the control group. 

### 2.3. Characterization

#### 2.3.1. Liquid Oxygen Compatibility Test

The liquid oxygen compatibility of materials can be determined by the mechanical impact in liquid oxygen ambient (LOC test). And the impact sensitivity can indicate the compatibility and stability of the material in LOX, to judge whether the material can be used safely in a liquid oxygen environment [33]. LOC tests of these polymer films (PCTFE, ETFE, FEP, PFA, PET and PI) were carried out according to ASTM G86-17. During the test, the observer recorded the phenomena of charring, flashing, explosion and combustion. Finally, the impact sensitivity coefficient (IRS) was used to evaluate the stability of the samples in LOX by recording the phenomenon of 20 impact tests. According to the test method, the LOC test of these polymer film materials before and after (60 days) immersion in LOX was completed, and the IRS was calculated using the following formula:

(1) $$\mathrm{IRS}=\frac{\sum_{i}^{4}w_{i}n_{i}}{N}\times 100\%$$

where $w_{i}$ is the weight coefficient and $w_{1}=1$ (combustion), $w_{2\;}=0.9$ (explosion), $w_{3}=0.6$ (flashing) and $w_{4\;}=0.4$ (charring). $n_{i}$ represents the frequency of the reaction.

#### 2.3.2. Cryogenic Tensile Test

**Figure 3:**
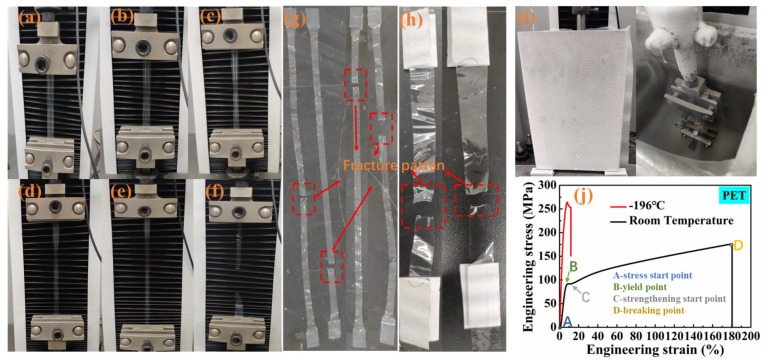
Specimen tensile process at room temperature (**a**–**f**), fracture specimens at room temperature (**g**), fracture specimens at low temperature (**h**), cryogenic tensile environment chamber (**i**) and tensile curve at room and low temperature (**j**).

The tensile test was performed on a universal material testing machine (WD-P3202, Jinan, Shandong, China) equipped with the liquid nitrogen environment chamber shown in Figure 3i. The sample was always immersed in LN_2_ during the whole tensile process. An initial tensile force of 0.5 N was applied to ensure that each specimen remained straight in the plane before running the stretching procedure. During the tensile process, the loading strain rate of each sample was 2 mm/min, and the procedure automatically stopped when the sample broke into two parts. As a result, the uniaxial tensile engineering stress–strain curve of each test specimen was gained by

(2) $$\sigma_{E}=\frac{F}{wt}$$

(3) $$\varepsilon_{E}=\frac{\Delta l}{l}$$

where $\sigma_{E}$, *F*, *w* and *t* are the engineering stress, the tensile force, the width and the thickness of testing specimen, respectively; and $\varepsilon_{E}$, Δl and *l* are the engineering strain, the deformation and the initial distance between two gauging lines, respectively. 

#### 2.3.3. Structural and Surface Characterizations

In order to further clarify the effect of liquid oxygen immersion on the structural and surface properties of polymer film materials, X-ray photoelectron spectroscopy (XPS, EscaLab-250 Xi, Thermo Fisher Scientific, Waltham, MA, USA) was employed to determine the surface element compositions before and after immersion in LOX. The attenuated total reflectance (ATR) spectrum (Nicolet IS50, Thermo Fisher Scientific, Waltham, MA, USA) was used to characterize the molecular structure changes during immersion. The surfaces of all specimens were studied by scanning electron microscopy (SEM, JSM-7610F, Hillsboro, OR, USA) equipped with an EDS analyzer. The EDS analysis was carried out in order to reveal the change of elements in the specimen surface. The effect of liquid oxygen immersion on crystallinity was investigated using X-ray diffraction (XRD, Empyrean, Almelo, Netherlands). And the relationship between the grain size and diffraction ray line broadening was given by Scherrer’s equation (Equation (4)), which was used to calculate the grain size [34,35].

(4) $$D=\frac{k\lambda }{\;\beta cos\theta }$$

where *D* is the crystal size, *k* is a constant, λ is the X-ray wavelength, *β* is the half-height width of diffraction peak and θ is the diffraction angle.

## 3. Results and Discussions

### 3.1. The Liquid Oxygen Compatibility of These Polymers

The impact sensitivity phenomena of these polymer films are listed in Table 1. It could be found that there were no phenomena in 20 impact tests for fluoropolymers, even after immersion for 60 days in LOX, which indicated that the four fluoropolymers were compatible with LOX, and the LOC could not be changed after long-time immersion in LOX. However, the IRS of PET and PI was 7.0% and 13.0%, respectively, which indicated that PET and PI were incompatible with LOX. However, the fluoropolymer coating could effectively reduce the impact sensitivity factor. According to the liquid oxygen impact test results, it could be concluded that fluoropolymer can be used safely in LOX, and the compatibility of PET and PI with LOX can be effectively improved by surface fluorination treatment.

### 3.2. Mechanical Properties

#### 3.2.1. The Effect of LOX Immersion on the Cryogenic Mechanical Properties

Before analyzing the effect of LOX on the cryogenic mechanical properties of polymers, we first analyzed the tensile tests of PET films at room and low temperature as an example of all film tensile processes. The process and results of the PET tensile tests are shown in Figure 3. It could be seen from Figure 3 of the PET films that the polymer film had good ductility at room temperature. Combined with the tensile curve, the whole process was divided into an elasticity stage (A–B), yield stage (B–C) and strengthening stage (C–D) in Figure 3j, corresponding to three tensile deformation processes, (a) to (b), (b) to (c) and (c) to (f) in Figure 3. The elastic deformation was reversible, and the (b) to (c) process belonged to the local deformation process. Then, during the (c) to (f) process, the deformation gradually expanded to both ends of the specimen with continuous stretching until finally breaking. From the fracture samples in Figure 3g, it could be seen that the deformation was irreversible. The whole cryogenic tensile process was completed in an ambient chamber, as shown in Figure 3i, during which the samples were immersed in LN_2_ until breaking. From the tensile curve in Figure 3j, it was found that compared with room temperature, the cryogenic tensile test could obtain a higher modulus and tensile strength at a great sacrifice of toughness. In addition, there was only an elastic stage and local yield stage in the cryogenic tensile process, and the tensile specimen broke in the process of (b) to (c) (local yield stage). Moreover, it could be seen from the fracture pattern in Figure 3h of cryogenic tensile specimens that there was obvious transverse deformation at the fracture, and it was not recoverable. Based on the above analyses, it was helpful for us to further study the properties of various polymer thin films at low temperature.

**Figure 4:**
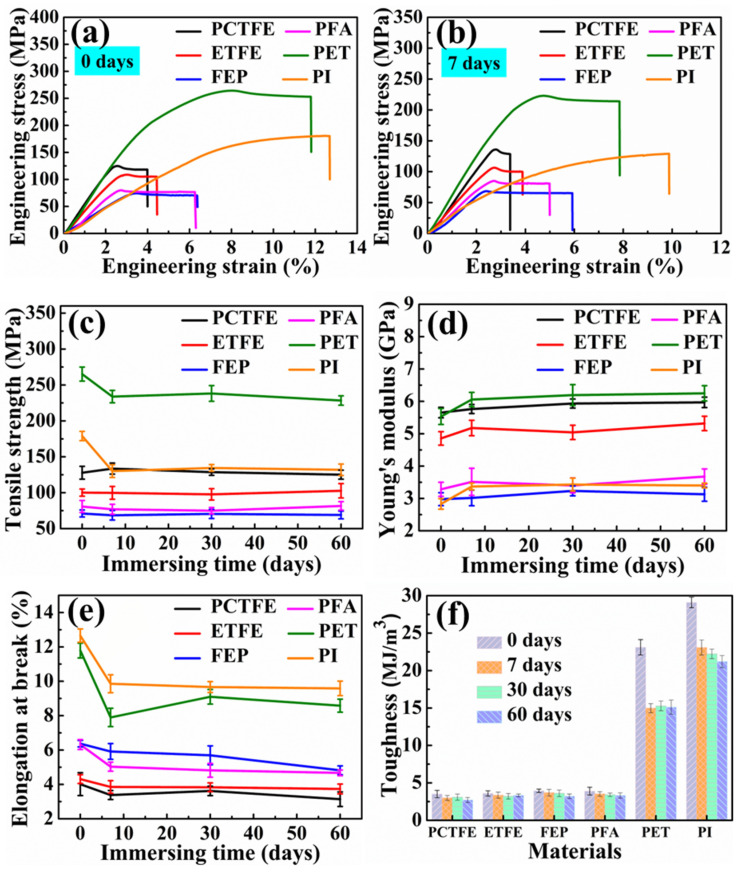
Cryogenic tensile stress–strain curves before (**a**) and after (**b**) immersion in LOX for 7 days, and tensile strength (**c**), Young’s modulus (**d**), elongation at break (**e**) and toughness (**f**) of these films under different immersion times.

In order to clearly show the effect of LOX on the cryogenic mechanical properties of these polymer film materials, the results of the tensile test and the variation trend of mechanical properties with the immersion time in LOX are shown in Figure 4.

As shown in Figure 4a, the cryogenic mechanical properties of fluoropolymer films and non-fluoropolymer films at 77 K were significantly different. Compared with the four fluoropolymer films, PET and PI possessed higher elongations at break at 77 K, and the cryogenic tensile strength of PET was higher than those of other polymer films. Among these fluoropolymers, PCTFE showed the highest tensile strength at 77 K, followed by ETFE. Though PFA and FEP showed lower strength, their elongation at break was higher than those of the other two fluoropolymers. In addition, Figure 4a,b show the tensile strength and the elongation at break of these polymer films with varying degrees of increase and decrease, respectively. In order to further analyze the changes, the variation trends of tensile strength, Young’s modulus and elongation at break of these polymer films at 77 K are plotted in Figure 4c–e, respectively, and the related data are shown in Table 2. Furthermore, the area under the stress–strain curves shown in Figure 4f is usually used to represent the fracture toughness of tensile materials [36,37,38].

It could be seen from Figure 4c that, compared to the untreated case, the cryogenic tensile strength of PET and PI obviously decreased after immersion for 7 days, but the decrease trend remained unchanged with the increase in immersion time. The related values are listed in Table 2. After immersion for 7 days, the cryogenic tensile strength of PET and PI decreased from the original 275.17 MPa and 178.98 MPa to 230.86 MPa and 130.05 MPa, reducing by 16.1% and 27.34%, respectively, while the cryogenic tensile strength of PCTFE, ETFE, FEP and PFA remained stable before and after immersion in LOX. As shown in Figure 4d, the cryogenic modulus of PET and PI increased clearly after immersion for 7 days, and then remained steady with the increase in immersion time. The cryogenic modulus values in Table 2 of PET and PI changed from 5.53 GPa and 2.83 GPa to 6.05 GPa and 3.39 GPa, increasing by 9.4% and 19.8%, respectively, after immersion for 7 days. However, for fluoropolymer film materials, the maximum change in cryogenic modulus after immersion for 7 days did not exceed 8.0%, and the degree of change was relatively small with the variation in immersion time. It could be found from Figure 4e and Table 2 that the elongation at break of PET and PI decreased most significantly after immersion for 7 days, which decreased by 33.0% and 23.7%, respectively. Then, no changes occurred in the elongation at break for PI after further immersion for 30 and 60 days, but there were small fluctuations for PET. The fracture toughness in Figure 4f exhibited the same variation trend as the elongation at break in Figure 4e, which decreased by 35.0% and 20.7% for PET and PI after immersion for 7 days, respectively, and then remained stable. For these fluoropolymers, the elongation at break shown in Figure 4e and the fracture toughness shown in Figure 4f all exhibited downward trends for PFA and FEP; however, they were more stable for PCTFE and ETFE. 

After the above analyses, the cryogenic mechanical properties of PET and PI obviously changed after immersion for 7 days in LOX, which might be related to the strong oxidation and ultra-low temperature of LOX. The amplitude of change indicated that PET and PI were more susceptible to the impact of the liquid oxygen environment compared with fluoropolymer materials. Nonetheless, the mechanical properties of PET and PI were always superior to fluoropolymer films at 77 K. For fluoropolymer films, the cryogenic tensile strength PCTFE > ETFE > PFA > FEP, while their cryogenic toughness exhibited the opposite trend.

#### 3.2.2. The Mechanical Properties after Immersion in LN_2_

**Figure 5:**
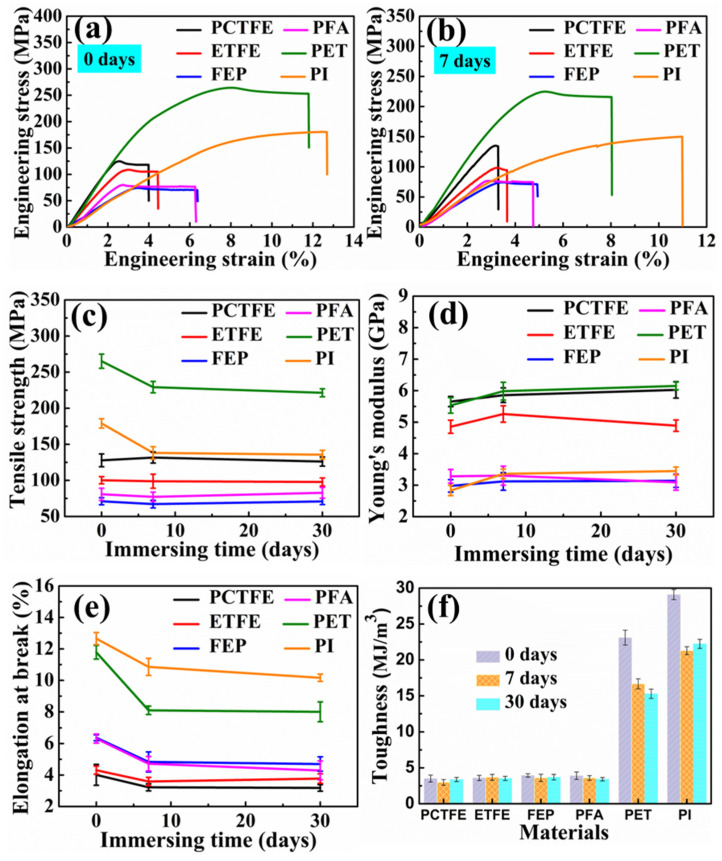
Cryogenic tensile stress–strain curves before (**a**) and after (**b**) immersion in LN_2_ for 7 days, and tensile strength (**c**), Young’s modulus (**d**), elongation at break (**e**) and toughness (**f**) of these films under different immersion times.

In order to distinguish the impact of the strong oxidation and ultra-low temperature on the mechanical properties of these film materials, the mechanical properties of these polymers after immersion in LN_2_ for 7 days and 30 days were investigated. Because of the few changes in mechanical properties during the later period of liquid oxygen immersion, the 60 day test might not be further explored. The test results are shown in Figure 5 and Table 3.

As can be seen from Figure 5, the cryogenic mechanical properties of these polymer films exhibited a consistent variation trend after immersion in LN_2_ and LOX. Figure 5c shows that the tensile strengths of PET and PI were more susceptible to the influence of ultra-low temperature, and the values in Table 3 were 229.24 MPa and 138.1 MPa, reducing by 16.69% and 22.84% after immersion for 7 days in LN_2_, respectively. However, the cryogenic strengths of the four fluoropolymers were stable, and the maximum value did not exceed 6%. The cryogenic modulus variation trend of these polymers in Figure 5d was similar to that in Figure 4d. Combining with the cryogenic modulus data in Table 2 and Table 3, there was little difference in the variation amplitude. Similarly, the elongation at break in Figure 5e and the fracture toughness in Figure 5f of these polymer films also decreased, which indicated that the cryogenic toughness of these materials became worse after immersion for a long time in LN_2_. Especially, for PET and PI after immersion for 7 days, the elongation at break reduced by 31.3% and 14.2%, respectively, and the fracture toughness reduced by 27.87% and 16.83%, respectively. The elongation at break of fluoropolymers reduced by 19.7% (PCTFE), 16.7% (ETFE), 22.6% (FEP) and 25.3% (PFA) after immersion for 7 days.

Based on the above analyses of the cryogenic mechanical properties of these polymer films after immersion in LOX and LN_2_, it can be concluded that the variation trends of cryogenic mechanical properties of the polymers after immersion in LN_2_ and LOX were basically consistent with the increase in immersion time. This indicated that the changes in cryogenic mechanical properties were largely related to the ultra-low temperature.

### 3.3. SEM Analysis

**Figure 6:**
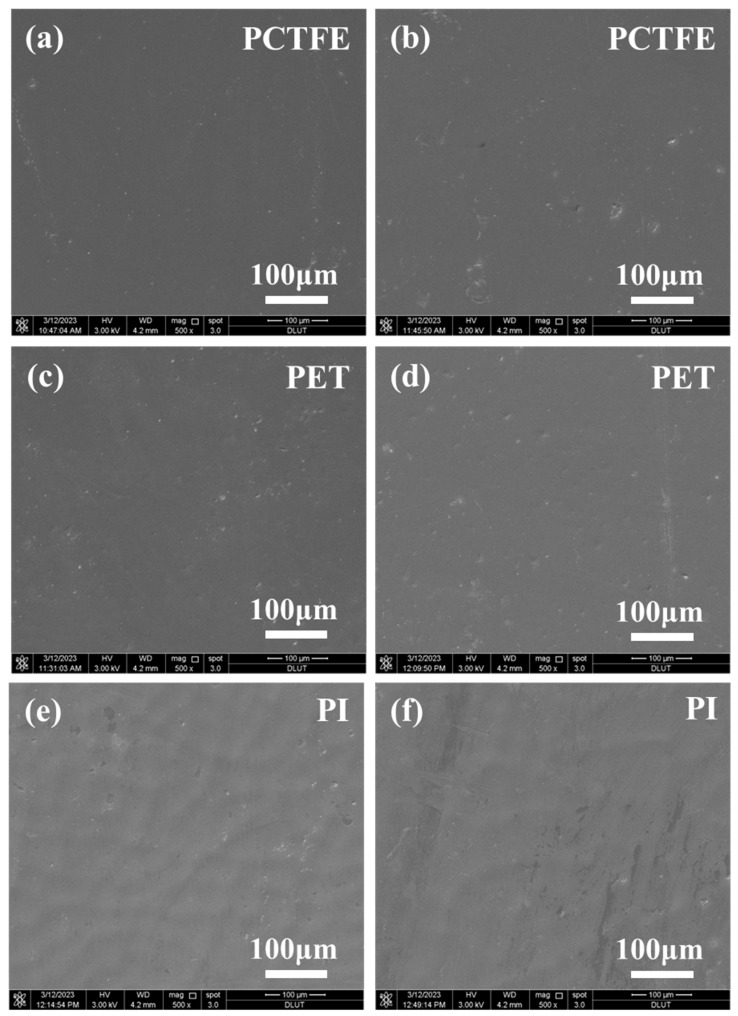
SEM images of PCTFE film, PET film and PI film sprayed with FEP powder before (**a**,**c**,**e**) and after (**b**,**d**,**f**) immersion in LOX for 60 days, respectively.

The tensile results of thin film materials are extremely susceptible to surface defects. Once there are defects on the surface of the sample, it is easy to generate stress concentration at the defects during the stretching process, which affects the test results. Therefore, SEM was employed to analyze the surface morphology of these material samples to further investigate whether defects had formed on their surfaces during immersion. Based on the above results of the cryogenic mechanical properties tests, PCTFE, PET and PI after immersion in LOX for 0 days (untreated) and 60 days were characterized. As shown in Figure 6, there were no defects on the surfaces of these polymer films after immersion for 60 days in LOX. The results of SEM indicated that long-term immersion in LOX could not cause defects on the surface of the films and the changes in cryogenic mechanical properties were not related to surface defects.

### 3.4. Chemical Structure Analysis

#### 3.4.1. XPS Analysis

**Figure 7:**
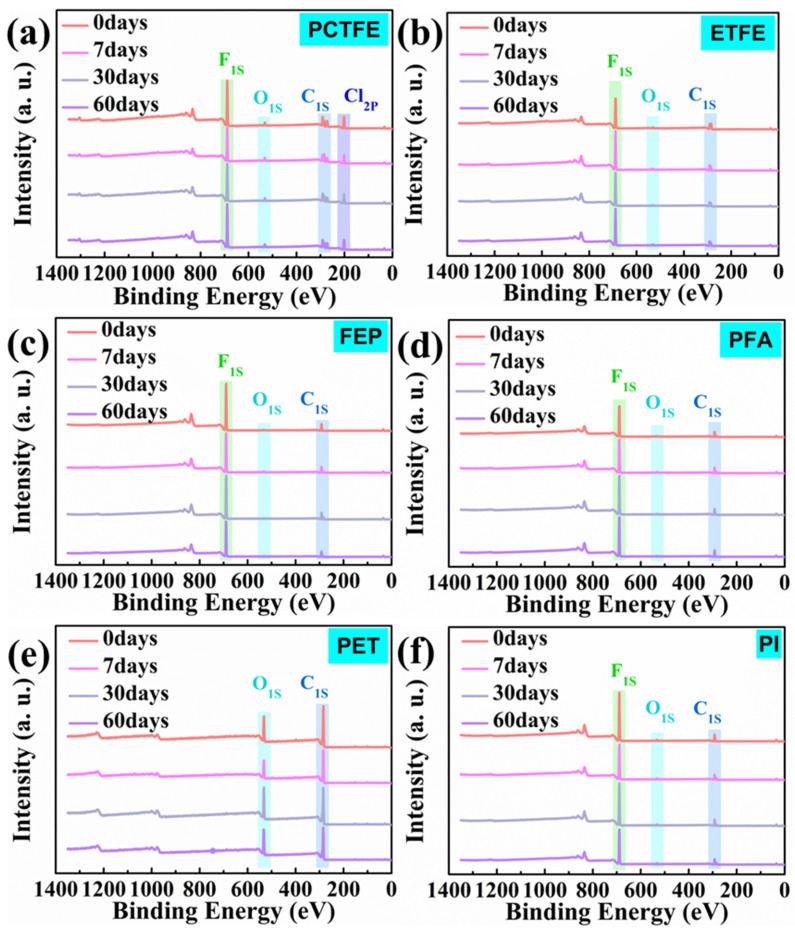
XPS spectra of PCTFE (**a**), ETFE (**b**), FEP (**c**), PFA (**d**), PET (**e**) and PI sprayed with FEP powder (**f**).

In order to further explore the relation between liquid oxygen immersion and the mechanical properties of these polymer films, XPS analysis was used to confirm the surface oxygen content of specimens before and after liquid oxygen immersion. Figure 7a–f show the XPS spectra of these polymer films before and after immersion. Due to the coating of FEP powder on the PI surface, its XPS spectrum was the same as that of FEP. For commercial-grade materials, the surface oxygen elements of the untreated samples correspond to the contamination [39,40]. The work of Nasef et al. [41] and Dasilva et al. [42] indicated that the pollution level could reach up to 2.7% or higher, so the oxygen content within this range can be considered to be caused by pollution. Table 4 records the oxygen content at different immersion times. The values in Table 4 show that the surface oxygen content of these materials remained steady after immersion for 7, 30 and 60 days. In addition, the molecular structure of PET contains oxygen, but its surface oxygen content basically remained stable with the change in immersion time.

**Figure 8:**
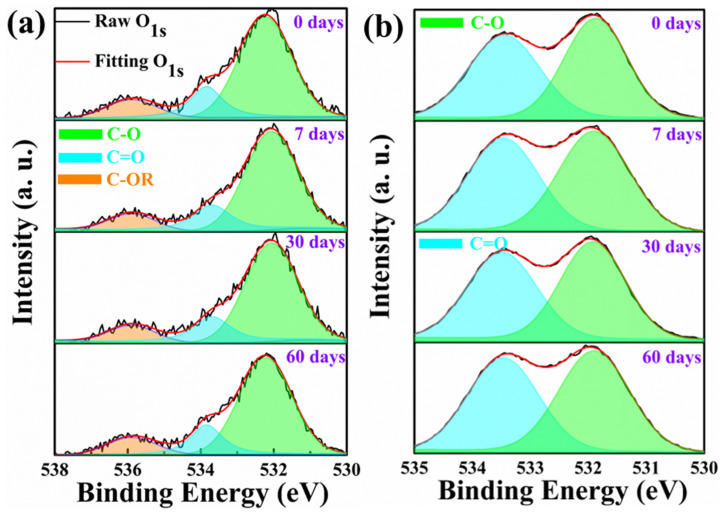
O_1s_ fitting peaks of PFA (**a**) and PET (**b**) (C-OR represents C-OC_n_F_2n+1_).

Since the molecular structures of PFA and PET contain oxygen elements, it is necessary to analyze their oxygen spectrum to further investigate whether the combination form of oxygen changed. The results are shown in Figure 8. From Figure 8a, it could be seen that the O_1s_ peak of PFA film was composed of three features: the main peak center at a binding energy of 532.4 eV, a low peak caused by pollution at 533.5 eV and another peak at 536.0 eV, corresponding to C-O, C=O and CF-O-C_n_F_2n+1_, respectively [43]. The O_1s_ spectrum of PET film showed two chemical states attributed to C-O (531.9 eV) and C=O (533.5 eV) [44,45] in Figure 8b. Figure 8 indicated that with the change in immersion time, there was no significant change in the absorption peak of the O_1s_ spectrum. In addition, the proportion of various bond areas of PFA and PET remained basically constant, which indicated that the oxygen bonding form of PFA and PET during immersion in LOX did not change. The results of XPS characterization showed that these polymer films did not undergo oxidation reactions during exposure to LOX for a long time, which also indicated that the changes in mechanical properties of these polymer materials were not related to the strong oxidation of LOX.

#### 3.4.2. EDS Analysis

EDS is also the most convenient technique for elemental identification and analysis. Table 5 shows the EDS test results. Comparing Table 4 and Table 5, it could be seen that the oxygen content measured using EDS was different to that using XPS, which might be caused by the different transmission depth of the two test methods. XPS is more susceptible to surface contamination [46]. In addition, the EDS results in Table 5 indicated that the oxygen content of these film surfaces after immersion for 7, 30 and 60 days did not significantly change. This further indicated that the changes in cryogenic mechanical properties of these polymer films were not related to the strong oxidation of LOX after immersion in LOX.

#### 3.4.3. ATR Analysis

**Figure 9:**
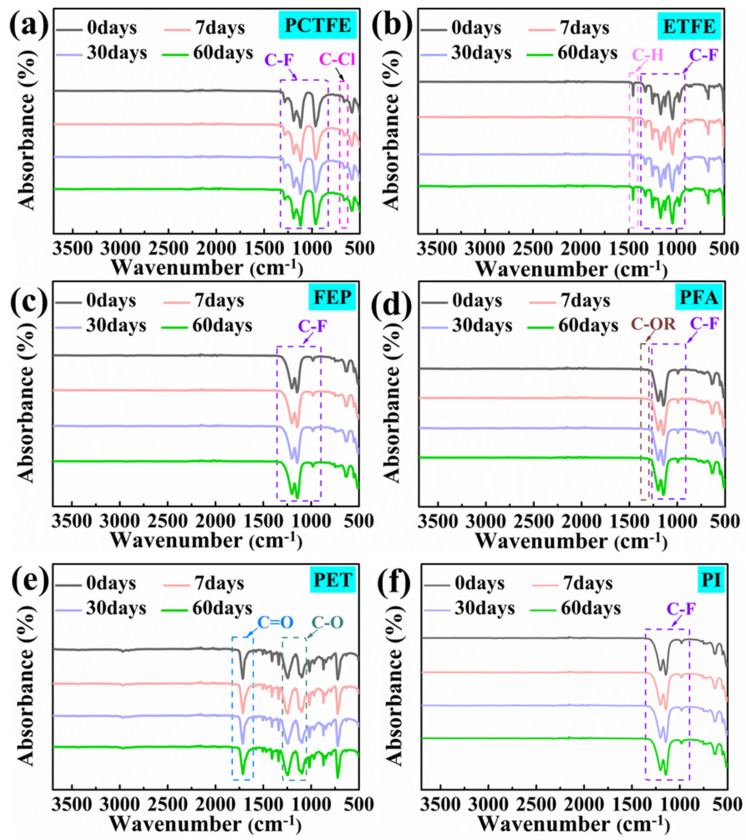
ATR spectra of PCTFE film (**a**), ETFE film (**b**), FEP film (**c**), PFA film (**d**), PET film (**e**) and PI film sprayed with FEP powder (**f**) (C-OR represents C-OC_n_F2_n+1_).

Figure 9 shows the ATR absorption spectra in the range of 500~3750 cm^−1^ of these polymer samples before and after immersion for different times in LOX. It is worth noting that the surface of PI film was coated with FEP powder, and there was no absorption band of the carbonyl group (C=O) on the spectrum in Figure 9f. This was also the reason why Figure 9c,f were similar. The peaks in the 1000~1400 cm^−1^ regions of the spectrum in Figure 9a–d, f are related to the C-F tensile vibrations; those in the 600~800 cm^−1^ regions in Figure 9a are related to the C-CL tensile vibrations; the sharp band at 1452 cm^−1^ in Figure 9b represents the C-H deformation; and there was a narrow sharp band at 1000~1150 cm^−1^ in Figure 9d, which represents the stretching vibration of the C-O group of the alkoxy vinyl [47,48,49,50]. In addition, the peaks in Figure 9e were identified at wavenumbers of 1710 cm^−1^ and 1000–1250 cm^−1^, corresponding to the ketone (C=O) and ether (C-O) bond [51,52], respectively. More importantly, it was observed that the characteristic peaks of these polymer samples did not change during the immersion process, and no new characteristic peaks occurred. Therefore, it could be further concluded that the surface chemical structure of these polymer films was stable and no oxidation reaction occurred during immersion in LOX.

Based on the above chemical structure analyses, it was known that these polymer films did not undergo an oxidation reaction when exposed to LOX for a long time. The results of these analyses also indicated that the changes in cryogenic mechanical properties were not related to the strong oxidation of LOX. This is not difficult to explain for fluoropolymers: their chemical stability in LOX stems from their strong antioxidant properties and good compatibility with LOX, which is why they are commonly used for manufacturing parts employed in liquid oxygen environments. The surface stability of PET in liquid oxygen environments might be related to the ultra-low-temperature environment of LOX. LOX provided an extremely low-temperature environment, thereby preventing polymer materials from being oxidized during immersion in LOX. It should be noted that PET and PI do not exhibit liquid oxygen compatibility, and they are prone to accidents due to external accidental load effects when used in liquid oxygen environments. Therefore, they cannot be directly used in liquid oxygen environments.

### 3.5. Crystal Phase Structure Analysis

#### 3.5.1. XRD Analysis after Immersion in Liquid Oxygen

In the previous section, we found that the changes in cryogenic mechanical properties were not related to the strong oxidation of LOX. However, there are studies that indicate that prolonged cryogenic treatment can affect the crystal structure of polymer materials, thereby affecting the mechanical properties [23,24,31]. Therefore, the XRD technique was used to characterize the crystallinity and size of crystalline particles, to further investigate the mechanism of the change in cryogenic mechanical properties of these polymer films after immersion in LOX.

**Figure 10:**
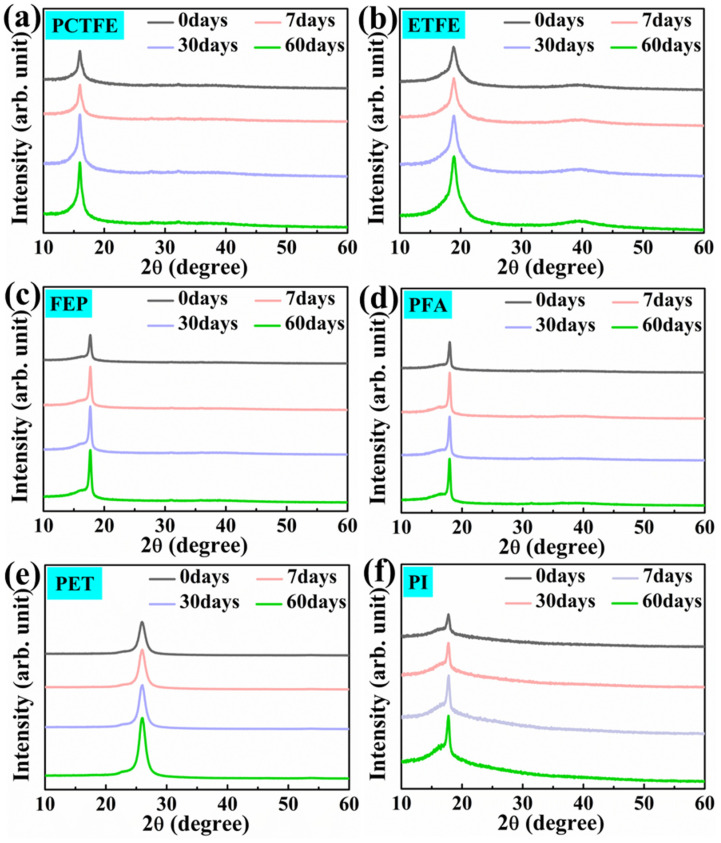
XRD spectra of PCTFE (**a**), ETFE (**b**), FEP (**c**), PFA (**d**), PET (**e**) and PI sprayed with FEP powder (**f**).

**Figure 11:**
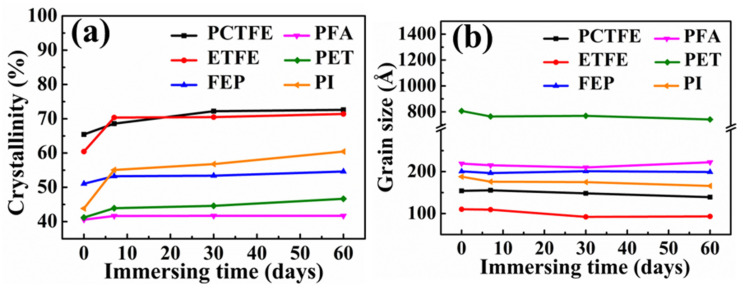
The crystallinity (**a**) and grain size (**b**) before and after immersion in LOX.

Figure 10 shows the XRD patterns of these polymers after immersion in LOX for different times. It can be seen from Figure 10a,c–f that these diffraction peaks at about 2θ = 17.8°, 2θ = 17.88°, 2θ = 17.9°, 2θ = 26° and 2θ = 18.3° corresponded to the crystalline phase regions of PCTFE, FEP, PFA, PET and PI [53,54,55,56,57]. However, there were two diffraction peaks at about 2θ = 19° and 2θ = 38° in Figure 10b, corresponding to the crystalline phase regions of ETFE [58,59]. With the increase in immersion time, the strength of these peaks of the crystalline region of these polymers increased, which indicates that the crystallinity of these polymer films might have changed after liquid oxygen immersion. In order to clearly draw a conclusion, the crystallinity of these polymers after different immersion times was calculated through MDI Jade analysis, as shown in Table 6 and Figure 11a. In addition, the calculation results of grain size using Scherrer’s equation are also given in Table 6 and Figure 11b. 

It could be seen from Figure 11a that the crystallinity of these specimens significantly increased after immersion for 7 days, and then tended to be stable, which was also consistent with the trend of changes in mechanical properties. In addition, the grain size of these polymers tended to gradually decrease as shown in Figure 11b, but the changes were not significant. These results indicated that when these polymer films were exposed to LOX for a long time, their crystallinity increased and their grain size was refined to varying degrees. At low temperatures, the level of atomic motion in polymers slowed down; however, it also increased the bond energy of the internal molecules, resulting in pure structural equilibrium throughout the material. Due to the influence of extremely low temperature, the bond length in the molecular chain decreased, and the bond angle orientation changed, thus resulting in changes in the crystal phase structure of the polymer materials, which was generally accompanied by the transition from the amorphous phase to the crystalline phase or the grain refinement of the crystalline phase. The polymer materials eventually reached an equilibrium state in internal structure at a corresponding low temperature, and showed an extremely fine, uniform and dense microstructure, which responded to the external loads with changes in mechanical and physical properties. Pande et al. [60] had calculated the model molecular structure at low temperature through quantum chemistry calculations and verified this phenomenon. In addition, some relevant papers had explained the crystal phase changes of polymer materials at low temperatures from the perspective of low-temperature relaxation [23,29]. They all believed that, compared with room temperature, even if the cooperative movement of the main chain movement of most polymers is greatly limited, local molecular movement still occurs. Molecules do move and form tighter, denser, rearranged patterns. Armeniades et al. [61] studied the mechanics and relaxation behavior of PET at low temperature through dynamic mechanics and stress–strain measurement, and they generally concluded that there was an obvious relaxation maximum at about 200 K. Furthermore, the dynamic mechanical measurements of crystalline and oriented PET specimens at cryogenic temperatures showed that the relaxation presented the maxima at 46 K and 26 K, respectively.

Therefore, the changes in mechanical properties of these polymers after immersion in LOX were largely caused by the changes in crystallinity and microcrystalline size. However, the changes in crystallinity and grain size were caused by the ultra-low temperature of LOX. Therefore, the fundamental reason for the changes in cryogenic mechanical properties of polymer films was the ultra-low temperature of LOX.

#### 3.5.2. XRD Analysis after Immersion in LN_2_

**Figure 12:**
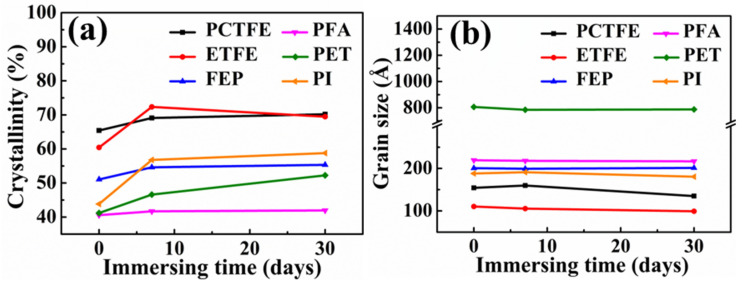
The crystallinity (**a**) and grain size (**b**) before and after immersion in LN_2_.

The XRD analysis was also conducted for these polymer films after immersion in LN_2_ for 7 and 30 days, and the results are shown in Figure 12 and Table 7. It could be seen from Figure 12 that the changes in crystallinity and grain size of these materials after immersion in LN_2_ were very similar to those after immersion in LOX. Moreover, the calculated values of crystallinity and grain size in Table 7 were basically consistent to the corresponding values in Table 6. Therefore, it could be further confirmed that the changes in cryogenic mechanical properties of these polymer films after liquid oxygen immersion were caused by the ultra-low temperature of LOX. 

## 4. Conclusions

In this paper, the LOC, the cryogenic mechanical properties and the performance stability of four fluoropolymer films (PCTFE, ETFE, FEP and PFA) and two non-fluoropolymer films (PET and PI) before and after immersion in LOX were investigated. And the following conclusions can be drawn:

(1) The four fluoropolymers were compatible with LOX and remained unchanged after immersion in LOX for 60 days. PET and PI exhibited incompatibility with LOX and could not be used in liquid oxygen directly. But their LOC could be improved by coating fluorine on the surface.
(2) Four fluoropolymer films and two non-fluoropolymer film materials still exhibited a certain strength and toughness at 77 K. Moreover, the cryogenic mechanical properties of PET and PI had always been superior to the four fluoropolymer film materials before and after immersion in LOX.
(3) With the increase in immersion time in LOX, the cryogenic mechanical properties of PET and PI were more easily affected, while the four fluoropolymers were relatively stable. In addition, the changes in cryogenic mechanical properties of these polymer films were caused by the changes in crystallinity and grain size because of the ultra-low temperature of LOX and were not related to the strong oxidation of LOX.
(4) Four fluoropolymers can be used as the inner layer materials of liquid oxygen composite hoses. PCTFE is the preferred material, followed by ETFE. In addition, due to the excellent cryogenic mechanical properties, PET and PI can be used as intermediate or outer layers after fluorine coating treatment. This work provides suggestions for the material selection process in the design and development of liquid oxygen composite hoses.

## Figures and Tables

**Table 1:** Impact sensitivity phenomena of several polymer materials in LOX.

Samples	Immersion Time (Days)	The Number of Impact Phenomena				The Total Number of Tests	IRS (%)
		Combustion	Explosion	Flashing	Charring		
PCTFE/ETFE/FEP/PFA	0/60	0	0	0	0	20	0
PET	0	0	0	1	2	20	7.0
PI	0	0	0	3	2	20	13.0
PI sprayed with FEP	0	0	0	0	2	20	4.0

**Table 2:** The cryogenic mechanical properties before and after immersion in LOX.

	Immersion Time (Days)	PCTFE	ETFE	FEP	PFA	PET	PI
Tensile strength (MPa)	0	127.78 ± 8.91	100.16 ± 5.03	71.08 ± 4.87	80.79 ± 8.43	275.17 ± 9.76	178.98 ± 6.44
	7	133.53 ± 7.72	99.76 ± 8.90	68.58 ± 6.64	76.90 ± 6.89	230.86 ± 8.63	130.05 ± 8.80
	30	128.67 ± 4.51	97.68 ± 7.83	70.47 ± 6.28	74.92 ± 4.54	238.24 ± 10.90	134.47 ± 4.73
	60	125.10 ± 6.33	102.68 ± 9.91	69.00 ± 5.37	81.59 ± 5.88	228.38 ± 6.54	131.87 ± 8.02
Elongation at break (%)	0	4.01 ± 0.66	4.31 ± 0.26	6.37 ± 0.19	6.32 ± 0.29	11.79 ± 0.43	12.65 ± 0.39
	7	3.38 ± 0.26	3.85 ± 0.36	5.92 ± 0.45	5.03 ± 0.26	7.90 ± 0.51	9.65 ± 0.52
	30	3.62 ± 0.27	3.83 ± 0.73	5.69 ± 0.55	4.82 ± 0.40	9.10 ± 0.42	9.60 ± 0.32
	60	3.14 ± 0.42	3.74 ± 0.30	4.8 ± 0.26	4.67 ± 0.16	8.58 ± 0.38	9.53 ± 0.43
Young’s modulus (GPa)	0	5.65 ± 0.16	4.85 ± 0.21	2.98 ± 0.20	3.28 ± 0.27	5.53 ± 0.25	2.83 ± 0.16
	7	5.76 ± 0.14	5.18 ± 0.24	3.02 ± 0.26	3.51 ± 0.42	6.05 ± 0.21	3.39 ± 0.10
	30	5.93 ± 0.18	5.04 ± 0.20	3.23 ± 0.15	3.40 ± 0.23	6.19 ± 0.32	3.43 ± 0.19
	60	5.97 ± 0.17	5.32 ± 0.22	3.13 ± 0.21	3.68 ± 0.24	6.24 ± 0.24	3.40 ± 0.07

**Table 3:** The cryogenic mechanical properties before and after immersion in LN_2_.

	Immersion Time (Days)	PCTFE	ETFE	FEP	PFA	PET	PI
Tensile strength (MPa)	0	127.78 ± 8.91	100.16 ± 5.03	71.08 ± 4.87	80.79 ± 8.43	275.17 ± 9.76	178.98 ± 6.44
	7	131.62 ± 7.74	98.83 ± 9.71	67.17 ± 5.33	77.23 ± 6.48	229.24 ± 7.91	138.1 ± 8.68
	30	126.10 ± 6.33	97.85 ± 5.49	70.91 ± 4.43	82.84 ± 7.42	221.38 ± 5.50	135.56 ± 6.03
Elongation at break (%)	0	4.01 ± 0.66	4.31 ± 0.26	6.37 ± 0.19	6.32 ± 0.29	11.79 ± 0.43	12.65 ± 0.39
	7	3.22 ± 0.25	3.59 ± 0.36	4.93 ± 0.64	4.72 ± 0.46	8.1 ± 0.27	10.86 ± 0.54
	30	3.19 ± 0.21	3.78 ± 0.33	4.70 ± 0.46	4.29 ± 0.60	8.0 ± 0.63	10.17 ± 0.23
Young’s modulus (GPa)	0	5.65 ± 0.16	4.85 ± 0.21	2.98 ± 0.20	3.28 ± 0.27	5.53 ± 0.25	2.83 ± 0.16
	7	5.86 ± 0.23	5.26 ± 0.26	3.12 ± 0.28	3.30 ± 0.28	5.98 ± 0.28	3.36 ± 0.15
	30	6.03 ± 0.26	4.89 ± 0.18	3.14 ± 0.21	3.09 ± 0.13	6.15 ± 0.12	3.45 ± 0.13

**Table 4:** The oxygen content on the surface of samples before and after immersion in LOX (XPS characterization).

Test Method	Sample	Oxygen Content (%)			
		0 Days	7 Days	30 Days	60 Days
XPS	PCTFE	3.70	3.53	4.13	3.61
	ETFE	1.27	1.96	1.47	0.61
	FEP	0.37	1.79	1.26	0.81
	PFA	1.65	2.17	1.63	0.50
	PET	21.62	18.11	25.48	24.14
	PI	4.83	5.66	4.96	5.71

**Table 5:** The oxygen content on the surface of samples before and after immersion in LOX (EDS characterization).

Test Method	Samples	Oxygen Content (%)			
		0 Days	7 Days	30 Days	60 Days
EDS	PCTFE	0.47	0.47	0.66	0.36
	ETFE	0.37	0.34	0.35	0.34
	FEP	0.32	0.35	0.31	0.31
	PFA	0.54	0.40	0.37	0.68
	PET	32.03	31.65	31.38	31.57
	PI	0.43	0.42	0.31	0.36

**Table 6:** The results from XRD tests before and after immersion in LOX.

Samples	Crystallinity (%)				Crystallite Size (Å)			
	0 Days	7 Days	30 Days	60 Days	0 Days	7 Days	30 Days	60 Days
PCTFE	65.44	68.59	72.20	72.61	154.23	155.47	148.23	139.16
ETFE	60.43	70.36	70.29	71.41	110.30	109.32	92.15	93.18
FEP	51.06	53.25	52.69	54.31	200.57	196.62	201.05	199.15
PFA	40.58	41.65	41.49	41.67	219.18	215.19	210.05	222.34
PET	41.21	43.92	44.61	46.66	801.17	783.99	788.79	780.82
PI	43.84	55.06	56.78	60.44	188.02	176.24	175.16	166.83

**Table 7:** The results from XRD tests before and after immersion in LN_2_.

Samples	Crystallinity (%)			Crystallite Size (Å)		
	0 Days	7 Days	30 Days	0 Days	7 Days	30 Days
PCTFE	65.44	68.14	70.18	154.20	159.79	134.92
ETFE	60.43	72.36	69.49	110.33	105.34	99.19
FEP	51.06	54.61	55.33	200.57	199.22	201.05
PFA	40.58	41.70	41.96	219.18	217.79	216.63
PET	41.21	46.61	52.25	801.17	784.45	787.64
PI	43.84	56.78	57.88	188.02	191.24	180.46

## Data Availability

The data presented in this study are available on request from the corresponding author.
